# Assessing Psychometric Properties of the Italian Version of the Heartland Forgiveness Scale

**DOI:** 10.3389/fpsyg.2020.596501

**Published:** 2020-12-17

**Authors:** Simone Consoli, Alessandro Rossi, Laura Y. Thompson, Clarissa Volpi, Stefania Mannarini, Gianluca Castelnuovo, Enrico Molinari

**Affiliations:** ^1^Psychology Research Laboratory, Ospedale San Giuseppe, Istituto di Ricerca e Cura a Carattere Scientifico (IRCCS), Istituto Auxologico Italiano, Verbania, Italy; ^2^Department of Philosophy, Sociology, Education and Applied Psychology, Section of Applied Psychology, University of Padua, Padua, Italy; ^3^Interdepartmental Center for Family Research, University of Padova, Padua, Italy; ^4^Independent Researcher, Phoenix, AZ, United States; ^5^Department of Psychology, Catholic University of Milan, Milan, Italy

**Keywords:** forgiveness, dispositional forgiveness, positive psychology, clinical and health psychology, confirmatory factor analysis, scale validation, construct validity, heartland forgiveness scale

## Abstract

Despite increasing popularity and intensive worldwide use, few studies have assessed the validity and factorial structure of the Heartland Forgiveness Scale (HFS). However, scientific literature showed that the original factorial structure of the HFS was not fully replicated and—in addition—the Italian translation is still lacking. To fill this gap, this study aims to extend evidence about the original HFS factorial validity by analyzing the Italian version. The final sample was composed of 523 randomly enrolled participants [139 males (26.6%), 384 females (73.4%)] aged from 18 to 82 years (mean = 42.53, *SD* = 16.41) who completed the Italian version of the HFS. The confirmatory factor analysis showed good fit indices for the original hierarchical factor solution and a significant decrease in model fit was found for all of the competing models. Also, the Italian version of the HFS revealed good reliability and very good psychometrical properties. Findings suggest that the Italian version of the HFS can be considered a reliable and good psychometrically based instrument for the assessment of dispositional forgiveness of the Self, Other, and Situation.

## Introduction

Over the past 30 years, the construct of forgiveness gained increasing attention and popularity allowing to produce a large amount of research in several fields (Freedman and Enright, 1996; Girard and Mullet, 1997; McCullough et al., 2000; Fehr et al., 2010; Wade et al., 2014; Akhtar and Barlow, 2016).

Forgiveness has a strong impact on psychological health and well-being of individuals (McCullough and Witvliet, 2002). Research showed a positive association between forgiveness (of self and/or others) and psychological health—reflected in the well-being indicators, namely, higher satisfaction with life and low levels of trait anger (Sells and Hargrave, 1998; Enright, 2001; Thompson et al., 2005b; Wade and Worthington, 2005; Walton, 2005). Moreover, forgiveness is related to lower psychopathological symptoms of anxiety and depression (Mauger et al., 1992; Hebl and Enright, 1993; Maltby et al., 2001; Baskin and Enright, 2004; Reed and Enright, 2006; Thompson et al., 2005a; Wade et al., 2005, 2014). Also, forgiveness is believed to represent an important factor in maintaining positive and healthy relationships with others (i.e., intimate relationships) (Mannarini et al., 2017). Conversely, non-forgiveness/unforgiveness may lead to a pessimistic view of the self, others, and the world, thus triggering or worsening depressive feelings (Beck, 1979; Hampes, 2016; Giuntoli et al., 2019).

Forgiveness is conceptualized as an overarching individual’s internal disposition to grant forgiveness, regardless of specific situation(s), and it may also be conceptualized as an adaptive trait, disposition, or behavior as well as a functional method of coping (Maltby et al., 2001). Forgiveness represents a way of responding to “transgression”—that is conceived as a negative event violating a person’s assumptions and expectations about people, the world, or oneself. These transgressions (real and/or perceived) could have an effect on the victim in so far as it may provoke negative thoughts (e.g., hostility), negative emotions (e.g., anger, sadness), and negative behaviors (e.g., revenge-seeking)—that in turn could lead to intense psychological distress. In this sense, the (dispositional) ability of one person to forgive may help to cope with the negative psychological sequelae of the transgression. Forgiveness allows to transform the subjective adverse responses—toward the transgression experience, the transgressor, or the transgression sequelae—from negative to neutral/positive (Thompson et al., 2005b).

Dispositional forgiveness is distinguishable according to the (potential) object of forgiveness—that is the source acting the transgression, for example, forgiveness of oneself for violating his/her own personal social and moral beliefs (e.g., guilt), and/or forgiveness of other specific(s) person(s) (e.g., injustice, offense), and/or forgiveness of situation that is beyond one’s control (e.g., illnesses, COVID-19, natural disasters, “fate”) (Thompson et al., 2005b). Thus, dispositional forgiveness is a complex construct and this intrinsic characteristic should be considered in its assessment.

To achieve this purpose and taking into account these different kinds of forgiveness, the Heartland Forgiveness Scale was developed (HFS; Thompson et al., 2005b). Considering the aforementioned background, the HFS is composed of three second-order factors, namely, “Self,” “Other,” and “Situation.” Furthermore, each of them is composed of two three-item first-order factors assessing the positive (forgiveness) and negative (unforgiveness) facets of their hierarchical dimensions: “Self-Positive,” “Self-Negative,” “Other-Positive,” “Other-Negative,” “Situation-Positive,” and “Situation-Negative.” Finally, positive and negative first-order factors also loaded onto independent second-order factors for “Positive” and “Negative” valence. Finally, in contrast with other self-report measures of forgiveness, the HFS does not refer to specific transgressions.

Given that a cross-cultural validation was strongly claimed (Thompson et al., 2005b; Fernández-Capo et al., 2017), the HFS has been translated worldwide into different languages, such as Arabic, Traditional Chinese, Filipino (Florendo et al., 2013), Greek, Indian (modified version; Dahiya and Rangnekar, 2018), Indonesian, Korean, Lithuanian, Persian, Polish, Portuguese, Slovak, Spanish, Thai, and Urdu. The Japanese version (Osanai and Furukawa, 2005), despite its good psychometrical properties, did not confirm the original factor structure, revealing two first-order latent dimensions: “self and situation” and “others.” In the Turkish version (Bugay et al., 2012), a first-order three-factor structure was found, confirming the existence of three scales reflecting forgiveness of the “self,” “others,” and “situations.” However, the Turkish version did not consider the aforementioned scales (self, other, and situation) as second-order factors—consequently neglecting the first-order factors suggested in the original validation (e.g., self-positive, self-negative, other-positive, etc.)—thus providing a simpler version and non-perfectly adherent to the original one.

However, only a few studies deeply investigated the HFS psychometric properties and even fewer assessed its structural validity (Osanai and Furukawa, 2005; Bugay et al., 2012). Furthermore, no one of them fully replicated the original HFS factorial structure—suggesting unclear results on its structural validity. Also, no questionnaire measuring dispositional forgiveness is currently available in Italian. Starting from this background, this study aimed to assess—for the first time—the factorial validity and the major psychometric proprieties of the Italian version of the HFS in an Italian sample.

## Materials and Methods

### Participants and Procedure

Several inclusion/exclusion criteria were applied. Inclusion were (A) being a native Italian speaker, (B) being over 18 years old, and (C) signing written and informed consent. Also, exclusion criteria were applied: (D) illiteracy and (E) impossibility to complete the questionnaire due to upcoming commitments and/or vision impairments.

Participants of each study were randomly recruited in Milan (Italy). Participants were randomly enrolled in Milan by means of personal invitations and advertisings in the University and in cafe bars and libraries in Milan. Moreover, the snowball sampling technique was used. The sample was composed of 523 participants.

According to guidelines (Beaton et al., 2000), the HFS was translated by two Italian expert psychologists and back-translated by an independent English translator to ensure cross-cultural equivalence. The Italian version of the HFS can be found at https://www.heartlandforgiveness.com/translations.

Participants completed the informed consent, a demographic measures form, and the Italian HFS. This research was approved by the Ethics Committee of Istituto Auxologico Italiano.

### Measure

The HFS is an 18-item questionnaire scaled on a 7-point Likert scale (from 1 = *“Almost Always False of Me”* to 7 = *“Almost Always True of Me”*) measuring dispositional forgiveness. The HFS consists of three major dimensions assessing (A) forgiveness of self [*Self—*items refer to negative emotions toward oneself (e.g., shame, guilt)], (B) others [*Other*—items refer to negative attitudes toward a transgressor (e.g., revenge)], and (C) situations [*Situation*—items refer to facing up to uncontrollable events (e.g., natural disaster, cancer)]. Each of these three major dimensions is composed of six items: three positively worded and three negatively worded, measuring forgiveness and unforgiveness, respectively (Thompson et al., 2005b). In this study, Cronbach’s alpha was (A) Self = 0.664, (B) Other = 0.604, and (C) Situation = 0.757.

### Statistical Analysis

Analyses were performed with R software (v.3.5.3) (R Core Team, 2017) by using corrplot (v.084) (Wei and Simko, 2017), psych (v. 2.0.7) (Revelle, 2018), and MplusAutomation (v.0.7-3) (Hallquist and Wiley, 2018) packages.

The MLMV estimator was used to perform the confirmatory factor analysis (CFA). Factorial validity was assessed by the Satorra–Bentler χ^2^ [*p*_(χ2)_ > 0.050 indicates a good model fit]. Moreover, goodness-of-fit indices were used with cutoffs criteria for ideal fit: RMSEA (<0.05); CFI (>0.90); SRMR (<0.08) (van de Schoot et al., 2012; Brown, 2015). Moreover, also two information criteria were computed: the AIC and the BIC.

According to the original study, six three-item first-order factors were specified for each of the positive (forgiveness) and negative (unforgiveness) factors of self, other, and situation. Moreover, these positive and negative first-order factors loaded onto their corresponding second-order—correlated—factors of Self, Others, and Situation. Finally, positive and negative first-order factors were also, respectively, specified as indicators of independent second-order factors for positive and negative valence.

Model comparisons were performed to exclude factorial structures different than the original one (Milavic et al., 2019; Rossi and Mannarini, 2019; Pietrabissa et al., 2020a). Specifically, the following were tested: (A) a single-factor model—specifying a single dimension called “Dispositional Forgiveness”; (B) a three-factor first-order model—specifying three simple first-order factors called “Self, Other, and Situation”; (C) a second-order model (hierarchical) with a general second-order factor and three first-order factors respectively called “Dispositional Forgiveness, Self, Other, and Situation”; and (D) a second-order model (hierarchical) retracing the hypothesized original model without “positive” and “negative” valence factors (graphical representation of these models were reported in the Supplementary Material 1).

Due to the non-nested nature of the aforementioned competing model, evaluations were performed by using differences in information criteria index: the model that showed the lower AIC and BIC was considered the best one (Cheung and Rensvold, 2002; Millsap, 2012; van de Schoot et al., 2012; Brown, 2015; Rossi and Mannarini, 2019).

Factor score determinacy coefficient (FS ≥ 0.7; good) (McDonald and Mulaik, 1979) was chosen as a measure of internal consistency of each single factor solution—both first- and second-order factors (Brown, 2003; Tabachnick and Fidell, 2014; Kline, 2016).

Moreover, the ability of the items to discriminate subjects with low or high dispositional forgiveness was tested. According to guidelines, (adjusted) item-total correlation was computed (Howell, 2013; Pallant, 2013; Tabachnick and Fidell, 2014). Moreover, considering the hierarchal structure of the HFS, two different “item discriminant power” (IDP) statistics—for typical performance items—were also carried out. Thus, IDPs were calculated both for first-order factors (Self-positive, Self-negative, Other-positive, Other-negative, Situation-positive, and Situation-negative) and for the second-order dimensions (Self, Other, and Situation). More in detail, for each considered factor, the total score and quartile rank for each subject was computed. Then, a series of independent-sample *t*-tests—and their effect size (Cohen’s *d*) (Cohen, 1988)—were computed to assess item discriminating power by using the total score of the scale as dependent variable and its lowest and highest quartile as grouping variable (Ebel, 1965; Chiorri, 2011).

## Results

### Sample Description

The sample was composed by 523 participants [139 males (26.6%), 384 females (73.4%)] aged from 18 to 82 years (mean = 42.53, *SD* = 16.41). Considering the civil status, the majority of the sample was single (207, 39.6%) followed by married people (180, 34.4%) or people who were in a relationship (70, 13.4%), and the others were separated (24, 4.6%), divorced (24, 4.6%), and widowed (18, 3.4%). Considering the education level, the majority of the sample had a bachelor/master degree (228, 43.6%), followed by people who had the high school license (186, 35.6%) and the remaining part of the sample had a middle school license (61, 11.7%), a Ph.D. degree (42, 8.0%), and the elementary school (6, 1.1%). Finally, considering the work status, the majority of the sample was a dependent worker (224, 42.8%), followed by entrepreneurs (93, 17.8%) and students (84, 16.1%); the remaining part of the sample was retried (75, 14.3%), unemployed (27, 5.2%), and housewife (20, 3.8%).

### Structural Validity

**FIGURE 1:**
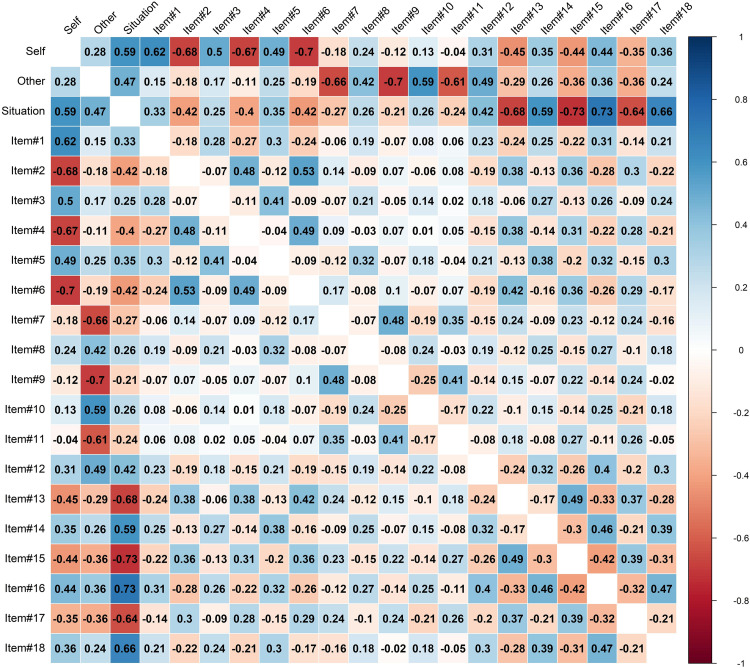
Item correlations.

As a preliminary analysis, the correlation matrix between the observed scores and the 18 items composing the HFS was computed (Figure 1)—suggesting the absence of excessive (positive or negative) relationships between the indicators (Tabachnick and Fidell, 2014; Brown, 2015).

**FIGURE 2:**
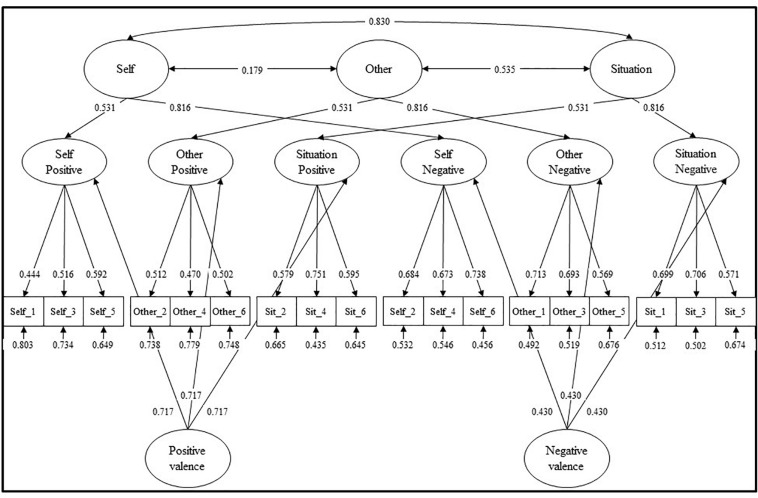
HFS structural model.

The CFA suggests a good solution for the original model: S-Bχ^2^ (133) = 223.95; *p* < 0.001; RMSEA = 0.036 [90% CI: 0.028–0.044; *p*(RMSEA = 0.05) = 0.998]; CFI = 0.935; SRMR = 0.066. Modification indices showed that the model could not be improved. Items factor loading ranged from 0.444 (item #1; “Self-Positive”) to 0.751 (item #16; “Situation-Positive”): *mean* = 0.612, *SD* = 0.09; with the items *R*^2^ ranging from 0.197 to 0.565: *mean* = 0.383, *SD* = 0.118. Finally, all second-order latent factor correlations were statically significant: *r*_(Self–Other)_ = 0.179; *r*_(Self–Situation)_ = 0.830; *r*_(Other–Situation)_ = 0.535. Results are shown in Table 1 and Figure 2.

**TABLE 1:** Item descriptive statistics, two different item discriminant powers (both on first-order and second-order factors; IDP 1st, IDP 2nd), and confirmatory factor analysis (CFA).

	**Descriptive statistics**						**IDP—on 1st-order factor**			**IDP—on 2nd-order factor**			**CFA**	
			
	**Mean**	***SD***	**Skewness**	**K**	**%Min**	**% Max**	***t***	***d***	***r* (it-tot)**	***t***	***d***	***r* (it-tot)**	**λ**	***R*^2^**

**SELF**														
**Positive**														
Item #1	4.75	1.733	–0.436	–0.480	6.5%	23.5%	–21.154	2.523	0.344	–15.715	1.879	0.395	0.444	0.197
Item #3	5.35	1.576	–0.999	0.662	4.2%	31.0%	–20.540	2.439	0.421	–12.094	1.421	0.278	0.516	0.266
Item #5	5.23	1.438	–0.814	0.776	3.1%	24.5%	–17.972	2.142	0.441	–10.910	1.298	0.285	0.592	0.351
**Negative**														
Item #2	3.70	1.840	–0.026	–1.002	19.3%	8.0%	–30.346	3.824	0.584	–18.436	2.201	0.462	0.684	0.468
Item #4	3.33	1.717	0.255	–0.757	21.8%	4.8%	–27.086	3.461	0.556	–17.866	2.134	0.475	0.673	0.454
Item #6	3.55	1.848	0.168	–0.977	20.5%	7.5%	–32.471	4.058	0.597	–20.915	2.487	0.490	0.738	0.544

**OTHER**														

**Positive**														
Item #8	4.89	1.427	–0.588	0.385	3.4%	15.3%	–15.018	1.940	0.271	–7.802	0.954	0.187	0.512	0.262
Item #10	4.21	1.723	–0.247	–0.623	10.7%	11.3%	–21.223	2.731	0.291	–13.881	1.695	0.350	0.470	0.221
Item #12	4.94	1.621	–0.497	–0.315	4.2%	23.5%	–17.858	2.313	0.256	–10.923	1.352	0.243	0.502	0.252
**Negative**														
Item #7	2.71	1.673	0.790	–0.137	34.6%	3.8%	–23.847	3.191	0.494	–18.251	2.275	0.441	0.713	0.508
Item #9	3.65	1.810	0.121	–0.936	17.0%	8.0%	–28.428	3.667	0.539	–20.153	2.423	0.481	0.693	0.481
Item #11	3.90	1.831	0.064	–0.889	13.4%	12.2%	–23.710	3.049	0.440	–17.645	2.154	0.359	0.569	0.324

**SITUATION**														

**Positive**														
Item #14	5.28	1.335	–0.635	0.627	1.9%	24.1%	–18.557	2.471	0.498	–13.548	1.659	0.432	0.579	0.335
Item #16	5.00	1.474	–0.411	–0.181	2.3%	21.4%	–24.661	3.285	0.556	–20.730	2.541	0.590	0.751	0.565
Item #18	4.75	1.676	–0.394	–0.517	5.4%	20.5%	–30.799	4.091	0.505	–18.755	2.306	0.466	0.595	0.355
**Negative**														
Item #13	3.61	1.765	0.160	–0.781	17.2%	7.8%	–26.205	3.371	0.511	–20.183	2.496	0.484	0.699	0.488
Item #15	3.52	1.625	0.116	–0.668	14.9%	4.8%	–24.622	3.073	0.530	–20.057	2.485	0.570	0.706	0.498
Item #17	3.33	1.764	0.282	–0.814	22.2%	5.7%	–24.662	3.173	0.440	–16.766	2.071	0.433	0.571	0.326

*All t-test and factor loadings (λ) are statistically significant at p < 0.001.*

Except for the “Negative valence” factor (FS = 0.510), all other reliability indices revealed an acceptable internal consistency of each factor solution: “Self-Positive”: FS = 0.823; “Self-Negative”: FS = 0.886; “Self”: FS = 0.777; “Other-Positive”: FS = 0.802; “Other-Negative”: FS = 0.860; “Other”: FS = 0.732; “Situation-Positive”: FS = 0.873; “Situation-Negative”: FS = 0.885; “Situation”: FS = 0.773; “Positive valence”: FS = 0.754.

### Model Comparison

The proposed original factorial structure solution was compared with different competing models that could also explain the HFS factorial structure (Muthén and Muthén, (1998-2012); Brown, 2015; Rossi and Mannarini, 2019). As reported in Table 2, model comparisons revealed the superiority of the original solution (Figure 2). Consequently, this factorial solution was chosen to perform after analyses.

**TABLE 2:** Model comparisons.

	**S-Bχ^2^ (*df*)**	**RMSEA**	**CFI**	**AIC**	**BIC**	**ΔAIC**	**ΔBIC**
Proposed model	223.950(133)***	0.036	0.935	34, 253.654	34, 492.191		
Model “A”	610.523(135)***	0.082	0.661	34, 842.983	35, 073.001	–589.329	–580.810
Model “B”	484.183(132)***	0.071	0.749	34, 652.112	34, 894.908	–398.458	–402.717
Model “C”	513.962(132)***	0.074	0.728	34, 692.764	34, 931.301	–439.110	–439.110
Model “D”	302.593(134)***	0.049	0.880	34, 370.929	34, 605.206	–117.275	–113.015

***p < 0.001. (A) a single-factor model—specifying a single dimension called “dispositional forgiveness”; (B) a three-factor first-order model—specifying three simple first-order factors called “Self, Other, and Situation”; (C) a second-order model (hierarchical) with a general second-order factor and three first-order factors, respectively, called “Dispositional Forgiveness, Self, Other, and Situation”; (D) a second-order model (hierarchical) retracing the hypothesized original model without “positive” and “negative” valence factors—this last model showed that the PSI matrix was not positive definite. AIC, Akaike information criterion; BIC, Bayesian information criterion; CFI, comparative fit index; RMSEA, root mean square error of approximation.

### Psychometric Properties

The IDP analysis showed that 18 items of the HFS discriminated well between subjects with low and high forgiveness of Self, Other, and Situation in both first-order and second-order dimensions (Table 1).

Considering the first-order dimensions: for the “Self-Positive” factor, the higher discriminative item was item #1 (*t*_*i*_ = −21.154, *p* < 0.001, *d* = 2.523); for the “Self-Negative” factor, the higher discriminative item was item #6 (*t*_*i*_ = −32.471, *p* < 0.001, *d* = 4.058); for the “Other-Positive” factor, the higher discriminative item was item #10 (*t*_*i*_ = −21.223, *p* < 0.001, *d* = 2.731); for the “Other-Negative” factor, the higher discriminative item was item #9 (*t*_*i*_ = −28.428, *p* < 0.001, *d* = 3.667); for the “Situation-Positive” factor, the higher discriminative item was item #18 (*t*_*i*_ = −30.799, *p* < 0.001, *d* = 4.091); finally, for the “Situation-Negative” factor, the higher discriminative item was item #13 (*t*_*i*_ = −26.205, *p* < 0.001, *d* = 3.371).

Considering the second-order dimensions, for the “Self” factor, the higher discriminative item was item #6 (*t*_*i*_ = −20.915, *p* < 0.001, *d* = 2.487); for the “Other” factor, the higher discriminative item was item #9 (*t*_*i*_ = −20.153, *p* < 0.001, *d* = 2.423); finally, for the “Situation” factor, the higher discriminative item was item #16 (*t*_*i*_ = −20.730, *p* < 0.001, *d* = 2.541).

In addition, the item-total correlation (adjusted) revealed statistically significant associations between each item and their respective first and second-order factors (Table 1).

## Discussion

The HFS is the first assessment tool evaluating dispositional forgiveness conceptualized as a multidimensional construct composed by the disposition to forgive self, the others, and the situation beyond one’s control (Thompson et al., 2005b). This study aimed at validating and analyzing the factorial structure of the Italian version of the HFS.

The results of this study showed that the Italian version of the HFS is a reliable assessment tool with a good structural validity. Moreover, this study replicates and confirms the original factor structure model proposed by Thompson et al. (2005b), including three distinct factors of dispositional forgiveness: self, others, and situations. In line with the view that forgiveness (measured by the HFS) is composed of three distinct, but interrelated, constructs of forgiveness of self, others, and situations, the forgiveness factors significantly correlated. The addition of “situations” as a source of transgressions (and as an object of forgiveness) differentiates the HFS from other forgiveness conceptualizations, previously including only forgiveness toward specific persons: the self or others.

The results showed that the HFS is suitable and useful for the assessment of dispositional forgiveness in Italian culture. Dispositional forgiveness is a wide and multifaceted construct without particular restrictions or limitations; it has been used worldwide and can be used in various research and clinical fields. For instance, the HFS is appropriate to assess the dispositional forgiveness level in psychological interventions and to monitor it over time—in the short and long term (Asper, 2016; Worthington and Sandage, 2016; Jackson et al., 2018). Properly assessing the dispositional forgiveness has important clinical implications. Indeed, a good assessment is the starting point to implement psychological interventions to promote dispositional forgiveness (e.g., forgiveness therapy or compassion therapy) to foster the treatment of psychopathological conditions and to improve the subjective well-being (Baskin and Enright, 2004).

This study is not free of limitations. Concerning the sample, the male and female proportion was unbalanced (73.4% females); future studies should deepen the gender differences in dispositional forgiveness. Despite a large sample allowing to correctly estimate the parameters used (*N* = 523), its size does not allow evaluating the measurement invariance both between males and females and across age. Thus, the sample could be further enlarged, possibly including particular clinical populations. Also, it should be highlighted that a high correlation between two latent factors, “Self” and “Situation” (*r* = 0.83), emerged. However, this coefficient is below the multicollinearity threshold (*r* = 0.84; Tabachnick and Fidell, 2014), and it should be noted that it is in line with previous literature, such as the original version by Thompson (*r* = 0.78; Thompson et al., 2005b) and the modified Indian one (*r* = 0.72; Dahiya and Rangnekar, 2018). To conclude, it should be highlighted the lack of convergent-divergent validity measures. Indeed, in the present study, no tools for the convergent-divergent validity were administered. Consequently, future studies should ascertain the relationships with other measures of dispositional and non-dispositional forgiveness as well as with other constructs (i.e., coping, acceptance).

Future research may explore dispositional forgiveness also in particular populations of persons and patients facing various psycho-physical issues. Forgiving a specific situation of physical illness (e.g., LVAD; COVID-19, cancer, etc.) (Mannarini et al., 2013; Ratti et al., 2017; Rossi et al., 2020; Parola, 2020; Parola et al., 2020; Rossi Ferrario and Panzeri, 2020) or a chronic (stressing) condition (e.g., aging difficulties, caregiving, obesity, dyadic conflicts) (Pietrabissa et al., 2017; Faccio et al., 2019; Panzeri et al., 2019; Balestroni et al., 2020; Panzeri and Rossi Ferrario, 2020; Parola and Felaco, 2020) may consistently help individuals to decrease denial and accept their situation, thus reducing the associated psychological distress (Elliott, 2011; Stuntzner and Dalton, 2015; Rossi Ferrario et al., 2019). Scientific literature showed that low levels of forgiveness may play a crucial role in patients with obesity who may show maladaptive behaviors, such as emotional eating and food addiction (Manzoni et al., 2020), as well as several related psychological issues (Mannarini and Boffo, 2014; Balottin et al., 2017; Manzoni et al., 2018; Simpson et al., 2018; Rossi and Mannarini, 2019; Pietrabissa et al., 2020b).

Among the strengths of this research, a robust and well-known statistical methodology was used. Noteworthy, this study provides the Italian version of the HFS and replicates—for the first time—the original second-order three-factor structure: all the other studies conducted in non-occidental countries found different factorial structures, thus suggesting a cross-cultural similarity between Italy and the USA. Future studies may assess the measurement invariance between Italy and the USA.

In conclusion, the Italian version of the HFS showed to be a good measurement tool with a good structural validity and reliability—allowing to assess dispositional forgiveness with its three dimensions of forgiveness toward self, others, and situations. Conducting a proper assessment of forgiveness is the first step to conduct effective and useful forgiveness-based psychological interventions to improve the psychological health of individuals both in clinical and other settings.

## Data Availability Statement

The raw data supporting the conclusions of this article will be made available by the authors, on reasonable requests.

## Ethics Statement

The studies involving human participants were reviewed and approved by the IRCCS Istituto Auxologico Italiano. The patients/participants provided their written informed consent to participate in this study.

## Author Contributions

AR and SC equally contributed and they share the first authorship. SC conceived the study and enrolled the sample. AR conceived the study, performed statistical analyses, wrote the first draft, and displayed tables and figures. CV helped with data collection. LYT, SM, GC, and EM provided important intellectual revisions.

## Conflict of Interest

The authors declare that the research was conducted in the absence of any commercial or financial relationships that could be construed as a potential conflict of interest.
