# Supplementary material for: Assessing Psychometric Properties of the Italian Version of the Heartland Forgiveness Scale
Source: Front Psychol. 2020 Dec 17;11:596501. doi: 10.3389/fpsyg.2020.596501 (PMC7773814; doi:10.3389/fpsyg.2020.596501)

S1. Supplementary material 1

Assessing Psychometric Properties of the Italian Version of the Heartland Forgiveness Scale

Simone Consoli[1], Alessandro Rossi[2,3*], Laura Y. Thompson[4], Clarissa Volpi[1], Stefania Mannarini[2,3], Gianluca Castelnuovo[1,5], Enrico Molinari[1,5]

[1]*Psychology Research Laboratory, Ospedale San Giuseppe, IRCCS, Istituto Auxologico Italiano, Verbania, Italy.*

[2]*Department of Philosophy, Sociology, Education, and Applied Psychology, Section of Applied Psychology, University of Padova, Padova, Italy.*

[3]*Interdepartmental Center for Family Research, University of Padova, Padova, Italy.*

[4] *Independent researcher, Phoenix, Arizona, USA*

[5] *Department of Psychology, Catholic University of Milan*

Confirmatory Factor Analysis (CFA) of the competing models of the Italian version of the Heartland Forgiveness Scale (HFS)

In order to test the goodness of the Italian version of the HFS, structural validity and graphical representation of the competing models tested were here reported.

Competing models were also described into the text (see the 'Statistical Analysis' section and the '*Model comparison*' paragraph in the 'Results' section).

Statistical analyses here reported were still performed with R software (v.3.5.3) (R Core Team, 2017) and the MplusAutomation (v.0.7-3) (Hallquist & Wiley, 2018) package. CFA were still performed using the same estimator – MLMV.

Model A

Structural validity

'Model A' was specified as a single-factor model in which each item loaded onto a single dimension called 'Dispositional Forgiveness'.

'Model A' showed a poor fit to the data. Indeed, the Chi-square statistic resulted to be statistically significant: S-Bχ^2 (135) = 610.523; $p < 0.001$; the RMSEA was equal to 0.082; 90%CI 0.076–0.089; p(RMSEA < 0.05) < 0.001; the CFI was equal to 0.661; and the SRMR equal to 2.629. As reported in Figure S1, items' loadings ranged from 0.250 (item#11) to 0.662 (item#16).

Figure S1 – graphical representation of 'Model A'

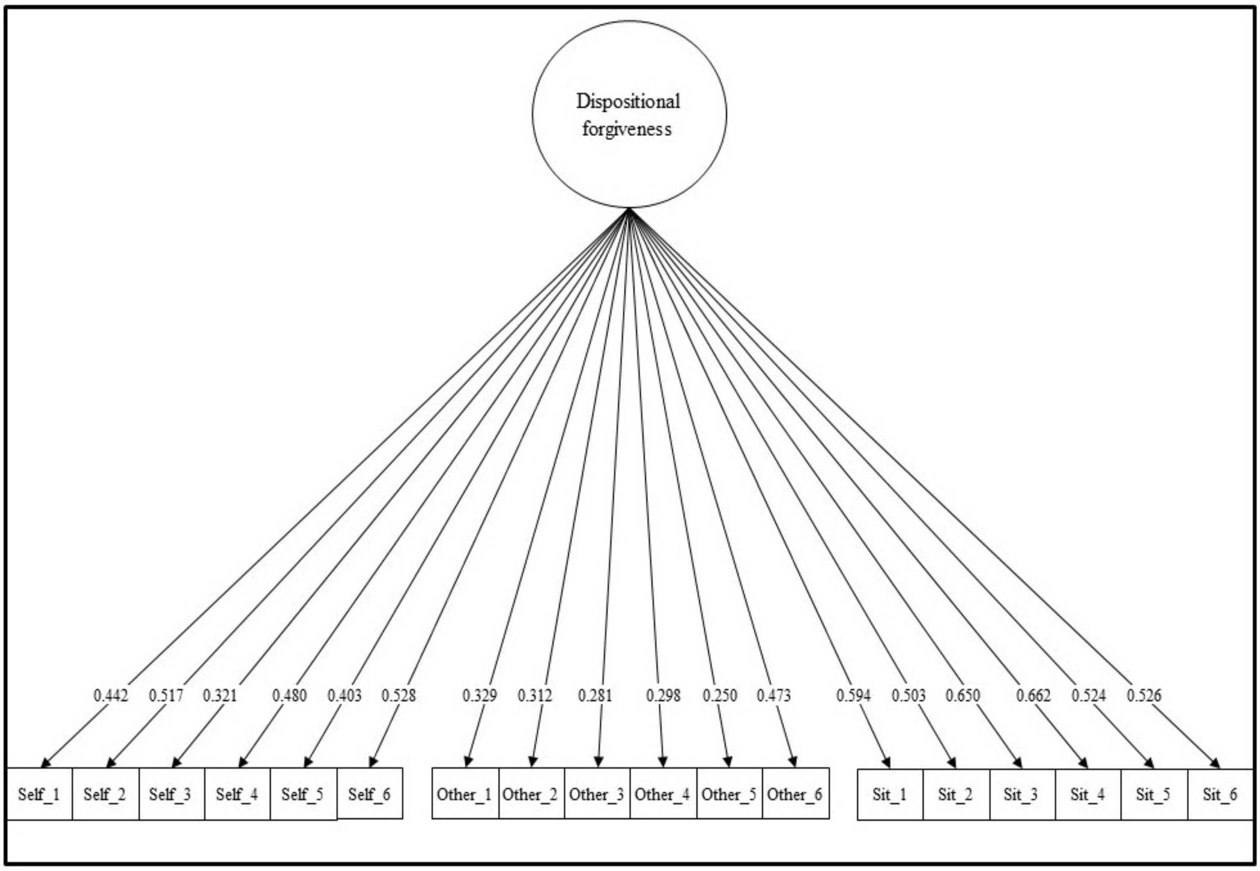

Model B

Structural validity

‘Model B’ was specified as a model with three-factor first-order model – namely, ‘Self, Other, and Situation’ – in which each item loaded onto its specific dimension.

‘Model B’ showed a poor fit to the data. Indeed, the Chi-square statistic resulted to be statistically significant: S-Bχ^2 (132) = 484.183; p < 0.001; the RMSEA was equal to 0.071; 90%CI 0.065–0.078; p(RMSEA < 0.05) < 0.001; the CFI was equal to 0.749; and the SRMR equal to 2.669. As reported in Figure S2, items’ loadings ranged from 0.217 (item#8) to 0.702 (item#6).

Figure S2 – graphical representation of ‘Model B’

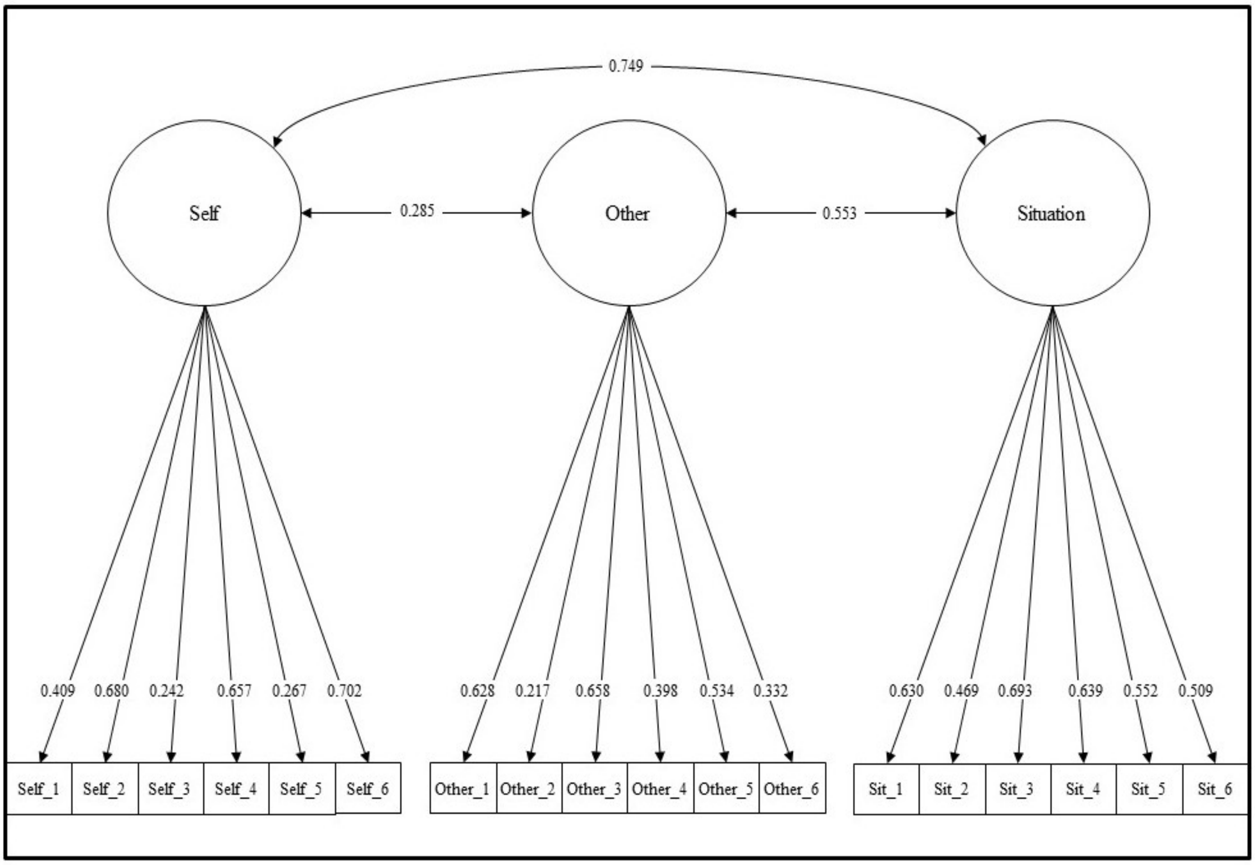

Model C

Structural validity

'Model C' was specified as a second-order model (hierarchical) with a general second-order factor and three first-order factors respectively called 'Dispositional Forgiveness, Self, Other, and Situation' – in which each item loaded onto its specific first-order factor which in turn it loaded onto the second order general latent dimension.

'Model C' showed a poor fit to the data. Indeed, the Chi-square statistic resulted to be statistically significant: S-Bχ^2 (133) = 513.962; $p < 0.001$; the RMSEA was equal to 0.074; 90%CI 0.067–0.081; p(RMSEA < 0.05) < 0.001; the CFI was equal to 0.728; and the SRMR equal to 3.159. As reported in Figure S3, items' loadings ranged from 0.215 (item#8) to 0.700 (item#15).

Figure S3 – graphical representation of 'Model C'

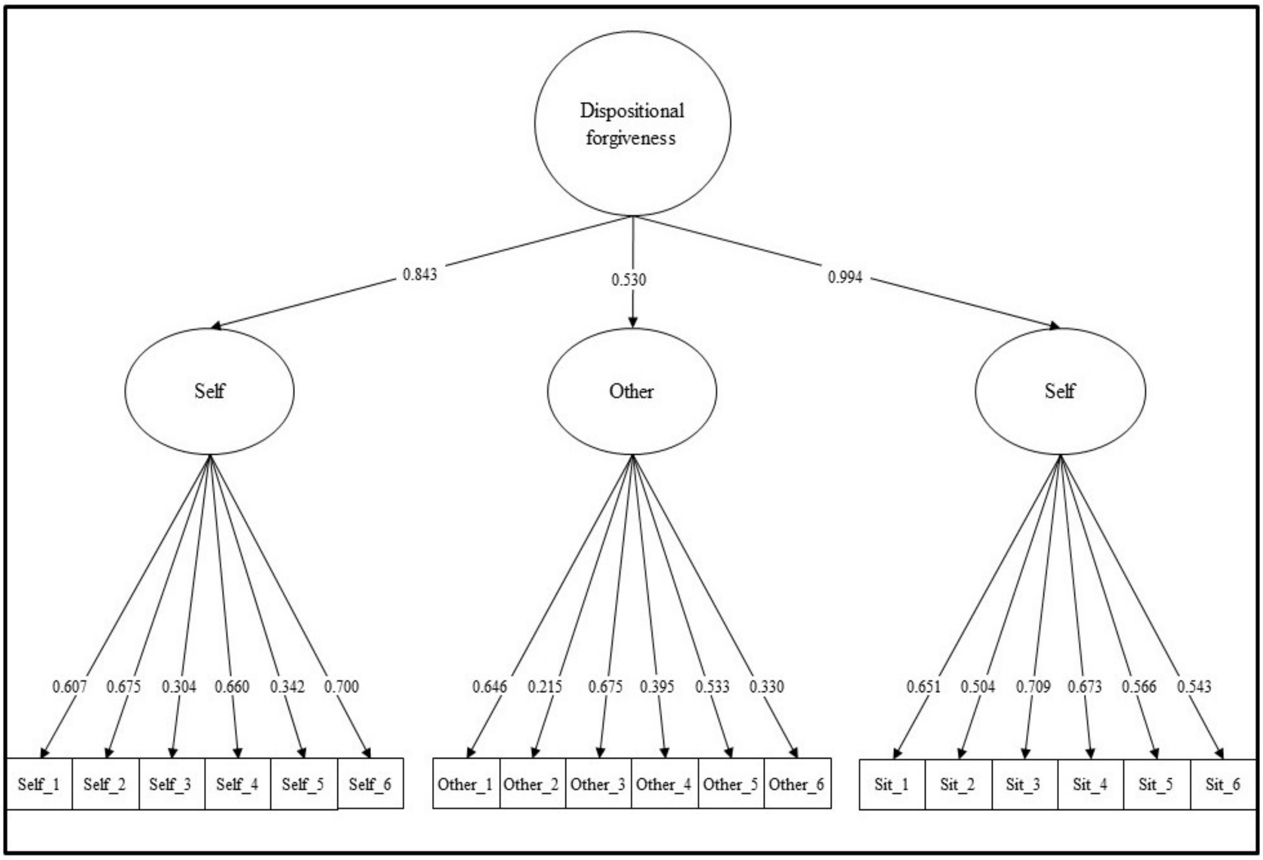

Model D

Structural validity

'Model D' was specified as a second-order model (hierarchical) retracing the hypothesized original model without 'positive' and 'negative' valence factors. More in detail, six three-item first-order factors were specified for each of the positive (forgiveness) and negative (unforgiveness) factors of self, other, and situation. Moreover, these positive and negative first-order factors loaded onto their corresponding second-order – correlated – factors of Self, Others, and Situation.

'Model D' showed a poor fit to the data and it revealed that the PSI matrix was not positive definite. The Chi-square statistic resulted to be statistically significant: S-Bχ^2 (134) = 302.593; $p < 0.001$; the RMSEA was equal to 0.049; 90%CI 0.042–0.056; p(RMSEA < 0.05) < 0.001; the CFI was equal to 0.880; and the SRMR equal to 2.239. As reported in Figure S4, items' loadings ranged from 0.461 (item#10) to 0.765 (item#16).

Figure S4 – graphical representation of 'Model D'

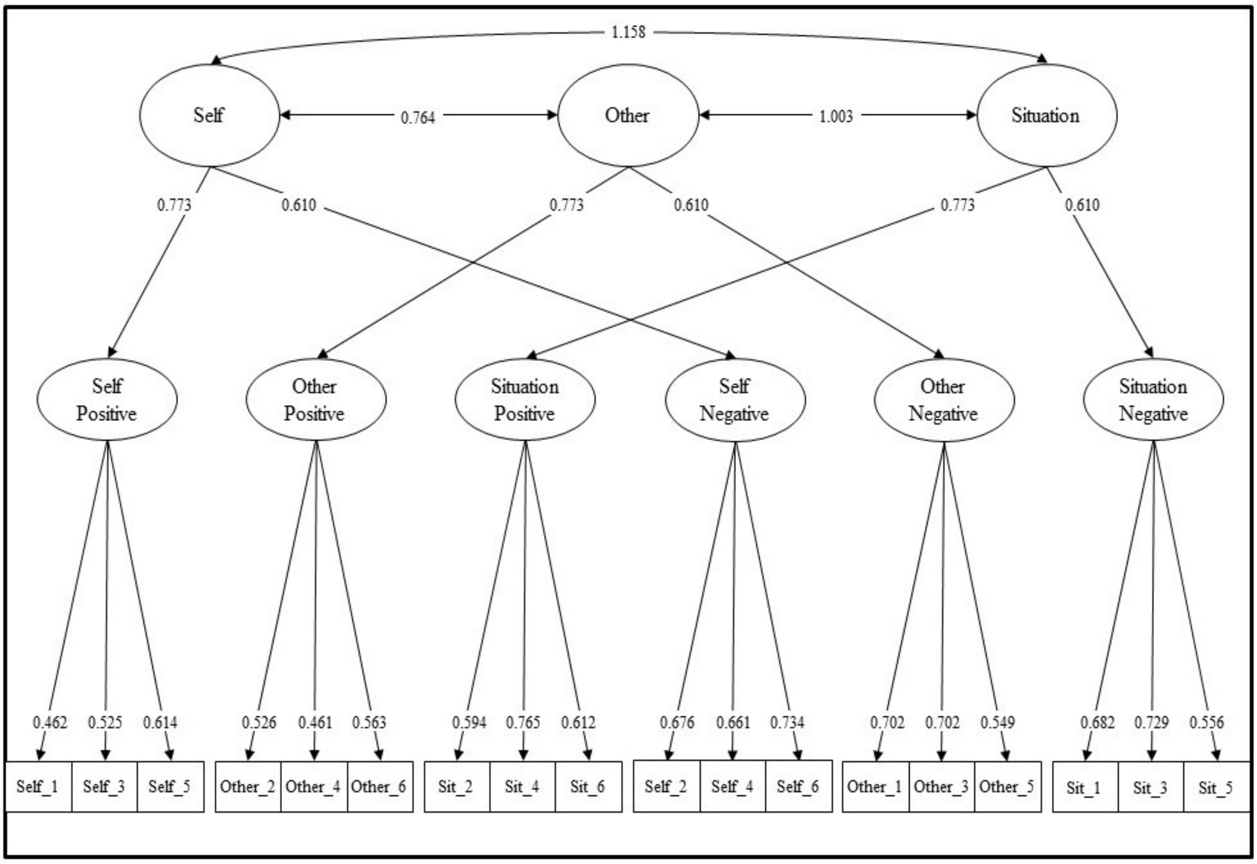

Supplement: Supplementary file 1 [file Data_Sheet_1.PDF]
